# Complete mitochondrial genome of *Iniistius trivittatus* and unique variation in two observed inserts between rRNA and tRNA genes in wrasses

**DOI:** 10.1186/s12862-020-01683-8

**Published:** 2020-09-21

**Authors:** Dong Liu, Yuanyuan Zhang, Ming Zhang, Jinquan Yang, Wenqiao Tang

**Affiliations:** 1 Shanghai Universities Key Laboratory of Marine Animal Taxonomy and Evolution, Shanghai, 201306 China; 2 Key Laboratory of Exploration and Utilization Aquatic Genetic Resources, Ministry of Education, Shanghai, 201306 China; 3 Shanghai Ocean University, National Demonstration Center for Experimental Fisheries Science Education, Shanghai, 201306 China; 4 Department of Epidemiology and Biostatistics, University of Georgia, GA, 30602 USA

**Keywords:** Fish, Molecular diversity, 16S rRNA, 12S rRNA, Transposon, Evolution

## Abstract

**Background:**

The family Labridae made up of 519 species in the world. The functional evolution of the feeding-related jaws leaded to differentiation of species, and the pharyngeal jaw apparatus evolved independently, but evolutionary mechanism still remain unaddressed in wrasses. Mitogenomes data can be used to infer genetic diversification and investigate evolutionary history of wrasses, whereas only eight complete mitogenomes in this family have been sequenced to date. Here, we sequenced the complete mitogenomes of *Iniistius trivittatus* to investigate genetic differentiation among wrasse species.

**Results:**

We sequenced the complete mitogenomes of *I. trivittatus* using a novel PCR strategy. The *I. trivittatus* mitogenomes is 16,820 bp in length and includes 13 protein -coding genes, 2 ribosomal RNA (rRNA) genes, 22 transfer RNA (tRNA) genes, and a control region. Compared to eight known mitochondrial genome, 2 additional noncoding regions (lengths of 121 and 107 bp), or so-called inserts, are found in the intergenic regions 12S rRNA - tRNA^Val^ - 16S rRNA. The presumed origin of the two rare inserts is from tRNA- related retrotransposons. Compared with cytochrome b gene, the two insert sequences are highly conserved at the intraspecies level, but they showed significant variation and low similarity (< 70%) at the interspecies level. The insert events were only observed in *I. trivittatus* by checking the phylogenetic trees based on the complete mitogenomes of Labrida species. This finding provides evidence that in the mitogenomes, retrotransposon inserts result in intraspecific homoplasmy and interspecific heteroplasmy by natural selection and adaptation to various environments.

**Conclusions:**

This study found additional mitogenome inserts limited in wrasse species. The rRNA genes with inserts might have experienced a selective pressure for adaptation to feeding modes. Such knowledge can enable a better understanding of molecular mechanism underlying morphological evolution in wrasses.

## Background

The Labridae family is the second largest group of marine fishes, and most species in this family inhabit the coastal and continental shelf waters near land to a depth of 200 m in tropical and temperate oceans. The family is divided into reefal and nonreef lineages, exhibits a high diversity of feeding ecologies, and plays a key role in sustaining reef environments [1]. There are approximately 519 known species of Labridae belonging to 71 genera in the world [2]. There are 150 species of labrid fishes within 38 genera in China, representing approximately 28.9% of the species worldwide [3]. The labrid family is diversified in shape, color, and size and includes many highly colorful species, several color patterns associated with sex and size, and some species that can change their sex from female to male [2]. In addition, hybrids have been described in labrid fishes [4]. These characteristics cause problems in species identification via morphological characteristics for some species.

Previous studies on labrid species have focused on their identification, istribution, behavior, and ecology. Some single-gene sequences or partial gene sequences have been used to reconstruct the phylogenetic relationships and evolutionary history of the labrid family [5]. Molecular analysis has revealed that the diversification of the labrid lineage is connected to its specialized pharyngeal jaw apparatus, which played an important role in enabling morphological evolution of the feeding apparatus in wrasses [6]. In the labrid family, the pharyngeal jaw bones of the paired lower jaw bones are united into a single jaw bone, which is suspended from the neurocranium and elevated by the muscular sling to crush and process food [7]. This special biting mechanism enables these fishes to feed on diverse prey, including gastropods, bivalves, crustaceans, fishes, coral mucous, zooplankton, ectoparasites, and algae [5]. Functional novelties in the feeding apparatus have allowed wrasses to occupy nearly every feeding guild in reef environments, which has led to diversification into various species.

**Fig. 1:**
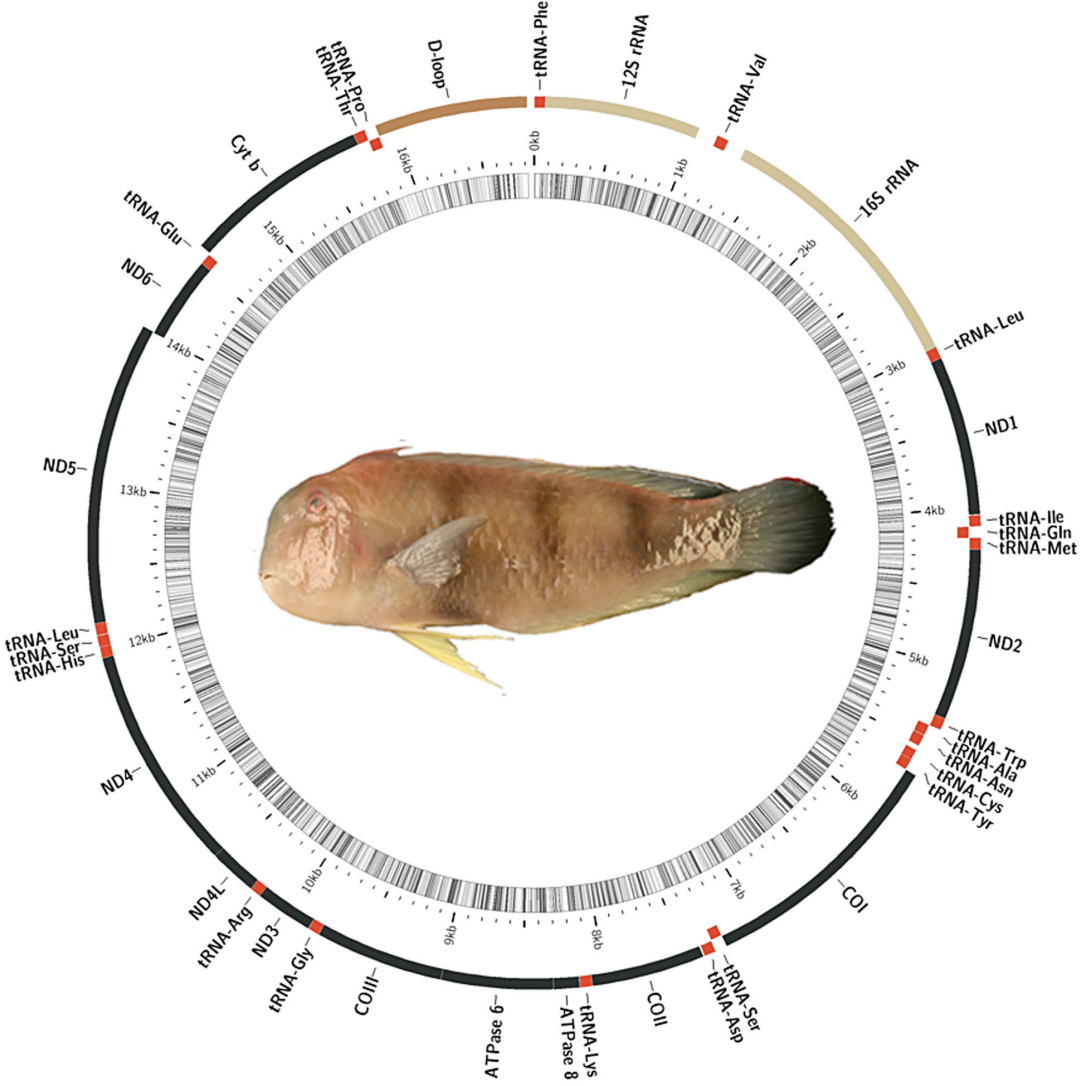
Graphical map of the mitochondrial genome of *Iniistius trivittatus*. Protein-coding genes, ribosomal RNA genes, and transfer RNA genes are shown using different colors. Genes encoded on the H-strand are in the outer region. Genes coded on the L-strand are in the inner region

The genus *Iniistius* in Labridae is represented by 21 named species worldwide [2], and 10 of these species are known to inhabit the reefs of China [3]. The species in this genus are popularly referred to as razorfishes due to their very compressed body and the firm, sharp ridge of their steep forehead and snout (Fig. 1). They live over open sandy bottoms and dive headfirst into the sand when predators approach [8]. *Iniistius trivittatus* has a limited distribution in the South China Sea near Hong Kong, Taiwan, and Guangzhou in China. Juveniles reside only in shallow water along coral reef edges on sand or rubble, and adults mainly reside in deep outer reef habitats [9]. This species differs from the other species of the genus *Iniistius*, a number of which have wide habitat ranges from the Atlantic Ocean to the Indo-Pacific Ocean and the Eastern Pacific Ocean [8], which leads to the question of how the various razorfish lineages have dispersed to achieve their present distribution. There is no evidence available based on the general structures and molecular markers that distinguish these species from other wrasses.

The mitochondrial genome of vertebrates contains 13 protein-coding genes, 2 ribosomal RNA (rRNA) genes, 22 transfer RNA (tRNA) genes, and a control region (CR) [10]. Mitochondrial DNA (mtDNA) has been widely used as molecular markers in taxonomy, population genetics, phylogenetics and evolutionary analysis due to its short length (15 − 17 k bp), maternal inheritance, fast rate of evolution, and rare recombination [11, 12]. mtDNA is under continuous natural selection because the 13 protein-coding genes produce polypeptide products [13], and strong positive selection has occurred on the mitochondrial gene *atp8* in phasianid birds for adaptation to the plateau environment [14]. The atp8 gene has also been noted as absent from the mitochondrial genomes of flatworms, nematodes, chaetognaths, rotiferans and bivalve molluscs [15]. The main noncoding region, the so-called control region or displacement loop (D-loop), is the most variable in vertebrate mtDNA and has been used extensively in studies of population genetics. A 40 bp insertion/deletion in the control region was found in sambar populations in India [16]. The length of the control region is highly variable in yellow-browed tit populations and in rice planthoppers due to the presence or absence of variable numbers of tandem repeats [17, 18]. Interestingly, in some plants, plastid DNA, acting as foreign DNA, can insert into the mitochondrial genome; the ribosomal protein genes in Alismatales show losses of one to six of the 14 ribosomal genes in the mtDNA [19]. The evolutionary history of mitochondrial genomes is astonishingly dynamic.

To date, there are only a few known complete mitochondrial genomes for the labrid family (no more than 8 species) [6, 20, 21]. Furthermore, there are no reports on the mtDNA characteristics of *Iniistius* species. Therefore, to elucidate the mitochondrial genome sequence of *I. trivittatus* and to provide molecular information for species identification, we sequenced and analyzed the complete mitochondrial genome of *I. trivittatus* with a novel strategy that includes three steps with six sets of primers for PCR and shotgun sequencing. Here, we report the complete mitochondrial genome of *I. trivittatus* and the presence of two additional noncoding region inserts, which may have originated from the insertions of transposable elements. We examined the polymorphisms in these inserts at the intraspecies and interspecies levels and found that these inserts are present in the genus *Iniistius* but not in the mtDNA of other wrasses. The data from this study will lead to a better understanding of the evolutionary patterns of the mitochondrial genome in the Labridae family and will help in species identification.

## Results

### Genome organization

The complete mitochondrial genome of *I. trivittatus* was sequenced, annotated, and deposited into the NCBI database (GenBank Accession MG976729). The mitochondrial genome was 16,820 bp in length. As expected, 37 typical mitochondrial genes, including 13 protein-coding genes, 12S rRNA, 16S rRNA, 22 tRNA genes, and a control region (D-loop), were identified (Fig. 1). Most of the genes were encoded on the H-strand, while 8 tRNA genes and ND6 were on the L-strand (Table 1). The *I. trivittatus* mitochondrial gene arrangement was identical to that of other mtDNAs [6, 20] and thus is highly conserved.

**Table 1:** Summary of the *Iniistius trivittatus* mitochondrial genome

Gene	Coding	Position		Size (bp)	Codon		Anticodon	Intergenic nucleotides
	Strand	From	To		Start	Stop		
tRNA (Phe)	H	1	68	68			GAA	
12S rRNA	H	69	1016	948				0
tRNA (Val)	H	1136	1209	74			UAC	119
16S rRNA	H	1316	2997	1682				106
tRNA (Leu)	H	2998	3071	74			UAA	0
ND1	H	3072	4043	972	ATG	TAG		0
tRNA (Ile)	H	4048	4116	69			GAU	4
tRNA (Gln)	L	4116	4186	71			UUG	-1
tRNA (Met)	H	4186	4255	70			CAU	-1
ND2	H	4256	5300	1045	ATG	T-		0
tRNA (Trp)	H	5301	5373	73			UCA	0
tRNA (Ala)	L	5376	5444	69				2
tRNA (Asn)	L	5446	5518	73				1
tRNA (Cys)	L	5550	5614	65				1
tRNA (Tyr)	L	5615	5684	70				0
CO I	H	5686	7236	1551	ATG	TAA		1
tRNA (ser)	L	7237	7307	71			UGA	0
tRNA (Asp)	H	7311	7382	71			GUC	3
CO II	H	7390	8080	691	ATG	T-		7
tRNA (Lys)	H	8081	8155	75			UUU	0
ATPase8	H	8157	8324	168	ATG	CAA		1
ATPase6	H	8315	8997	683	ATG	TA-		0
CO III	H	8998	9782	785	ATG	TA-		0
tRNA (Gly)	H	9783	9853	71			UCC	0
ND3	H	9855	10,206	352	ATG	T-		1
tRNA (Arg)	H	10,207	10,275	69			UCG	0
ND4L	H	10,276	10,572	297	ATG	TAA		0
ND4	H	10,566	11,946	1381	ATG	T-		−6
tRNA (His)	H	11,947	12,015	69			GUG	0
tRNA (Ser)	H	12,016	12,084	69			GCU	0
tRNA (Leu)	H	12,089	12,160	72			UAG	4
ND5	H	12,162	14,006	1845	ATG	TAA		1
ND6	L	14,003	14,524	522	ATG	TAA		−3
tRNA (Glu)	L	14,525	14,593	69			UUC	0
Cyt b	H	14,605	15,745	1141	ATG	T-		11
tRNA (Thr)	H	15,746	15,816	71			UGU	0
tRNA (Pro)	L	15,816	15,885	70			UGG	−1
Control region	H	15,886	16,820	935				0

The *I. trivittatus* mtDNA nucleotide composition is 25.6% T, 30.1% C, 26.6% A, and 17.7% G. The overall content of A + T is 52.2%, which is slightly richer than that of C + G (47.8%). Overall, the mtDNA showed AT- and GC-skew values of 0.019 and − 0.259, respectively, suggesting quite similar numbers of A and T nucleotides and a strong excess of C over G nucleotides, indicating strand compositional bias.

### Protein-coding genes

Twelve of the 13 *I. trivittatus* protein-coding genes were encoded on the H-strand, and only ND6 was encoded on the L-strand. All of the genes were initiated with the start codon ATG, and the following types of complete termination codons were observed: TAG (ND1), TAA (COI, ND4L, ND5, and ND6), and CAA (atp8). Seven of the protein-coding genes had incomplete stop codons (T- or TA-). The two regions ND4-ND4L and ND5-ND6 had 6 and 3 nucleotide overlaps, respectively. Intergenic regions with spanning sequences of 1–11 nucleotides were found in six protein-coding genes (COI, COII, atp8, ND3, ND5, and Cyt b) and their flanking tRNAs (Table 1).

### RNA genes

**Fig. 2:**
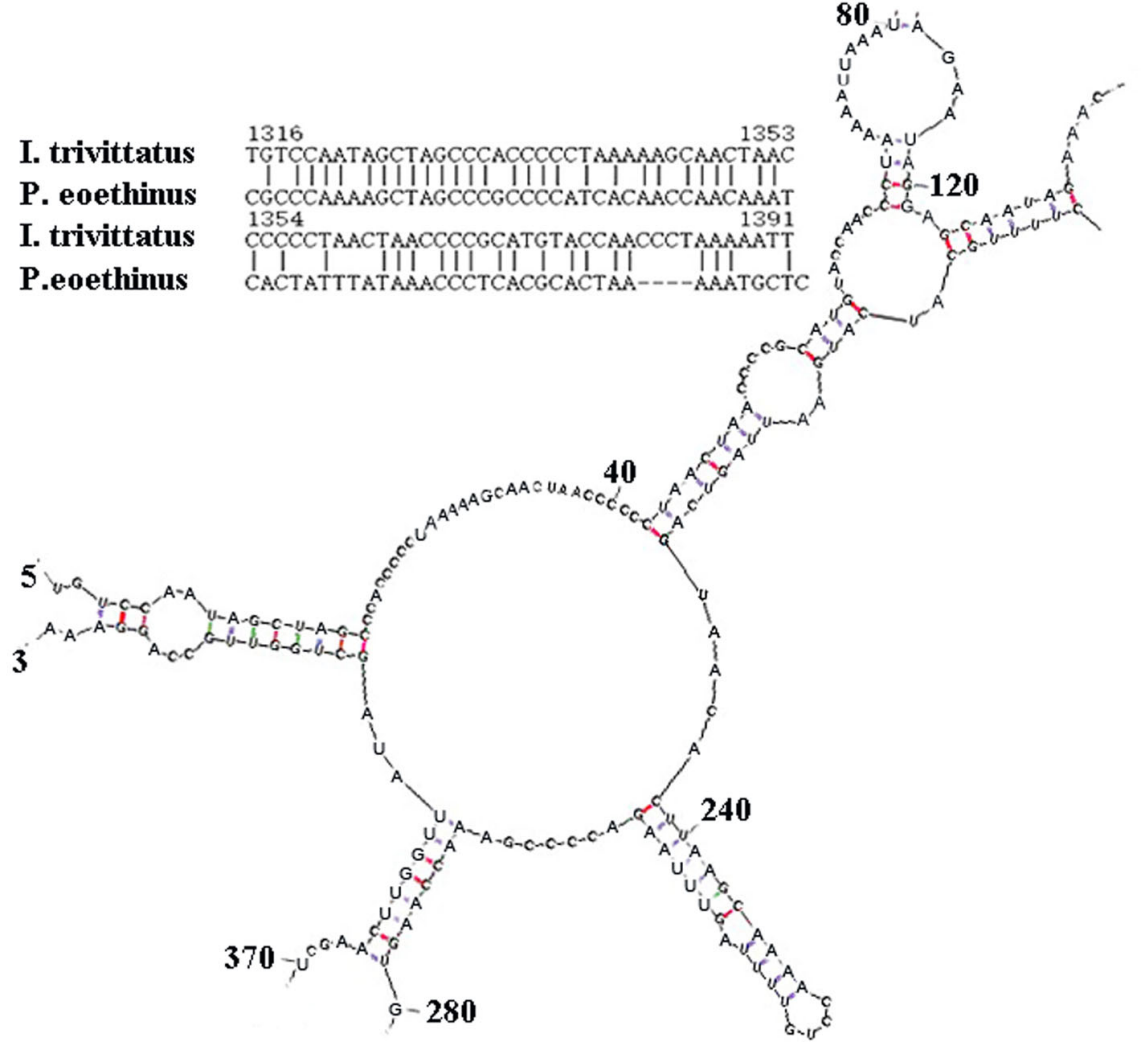
The secondary structure of the 5′ end of the mitochondrial 16S rRNA gene for *Iniistius trivittatus* (partial diagram) and the location initiated from the 5′ end of the structure diagram. The aligned sequences for *I. trivittatus* and *Pseudolabrus eoethinus* are shown for only a short portion of the 5′ end of the 16S rRNA gene, and the number indicates the site in the complete mitochondrial genome

The 22 identified tRNA genes ranged from 65 to 75 bp long. Among these genes, 14 tRNA genes were encoded on the H-strand, and eight were located on the L-strand. All of the tRNA genes were predicted to fold into the expected cloverleaf secondary structure. The two rRNA genes, 12S rRNA and 16S rRNA, were 948 and 1682 bp long, respectively. The 12S rRNA gene was located between the tRNA^Phe^ and tRNA^Val^ genes, and the 16S rRNA gene was located between the tRNA^Val^ and tRNA^Leu^ (UAA) genes. Both of the 400 bp long ends of each of the two rRNA genes can be folded into typical secondary structures, which is similar to patterns in other wrasses, such as *P. eoethinus* (GenBank Accession EU560728), and shows the base pairing of helices. For example, although the similarity of the 16S rRNA 5′ end sequences is very low between *I. trivittatus* and *P. eoethinus*, their 400 bp ends can be folded into similar secondary structures (Additional Figs. 1 & 2), and most of the unmatched nucleotides situated in single strand did not affect the secondary structures. A conserved motif (AGCTAGCCC) was found in the first stem structure that can serve to identify the 5′ start sequence of the 16S rRNA gene (Fig. 2).

### Noncoding region

**Fig. 3:**
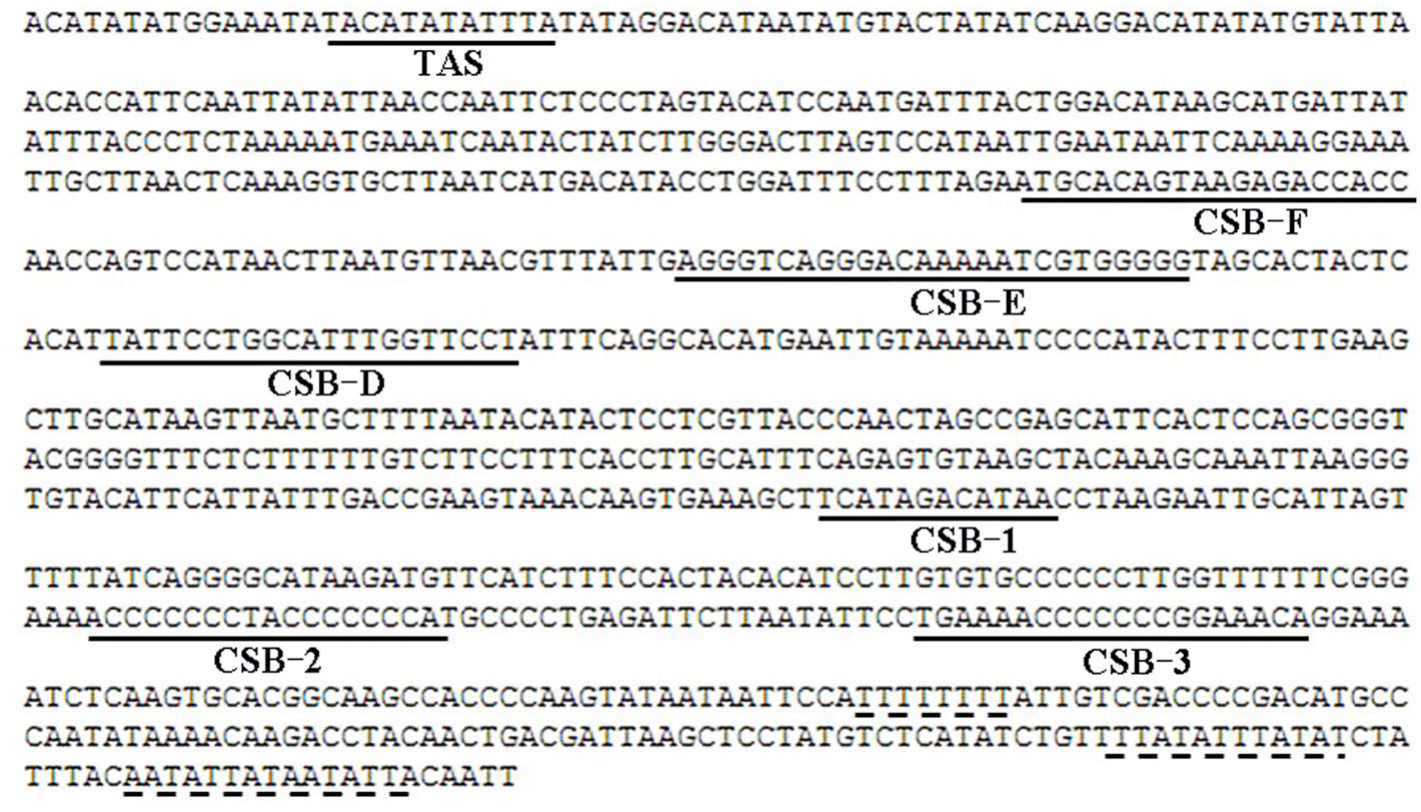
Sequence of the mitochondrial control region in *Iniistius trivittatus*. The termination-associated sequence (TAS), central conserved sequence blocks (CSB-F, CSB-E and CSB-D), and conserved sequence blocks (CSB-1, CSB-2 and CSB-3) are underlined, and the blocks composed of repeat elements are indicated by dashed lines

As a common noncoding region, the control region in the mtDNA of *I. trivittatus* was 935 bp long, and three conserved domains were identified by sequence alignment with other mtDNAs: the termination associated sequence (TAS), central conserved sequence blocks (CSB-D, CSB-E, and CSB-F), and conserved sequence blocks (CSB-1, CSB-2, and CSB-3) (Fig. 3). The motifs within these conserved domains were identified by similarity analysis and were as follows: a 12 bp long motif in the TAS domain, a 21 bp long motif in the CSB-D domain, a 20 bp long motif in the CSB-E domain, and a 20 bp long motif in the CSB-F domain. The origin of mitochondrial DNA replication, the CSB-1 domain, which is variable in most vertebrates, was found to have a 12 bp long motif. The CSB-2 domain, with a motif of two nucleotides (TA) and its flanking strings of C, was identified. A motif characterized by ~ 80% AC content was present in the CSB-3 domain. Additionally, there were three types of repeat elements: a poly (T) stretch of 8 bp, a “TTTATA” unit with 2 copies, and a “AATATTA” unit repeated once immediately after 1 bp in the 3′ portion of the conserved sequence blocks.

### Additional inserted noncoding regions

**Fig. 4:**
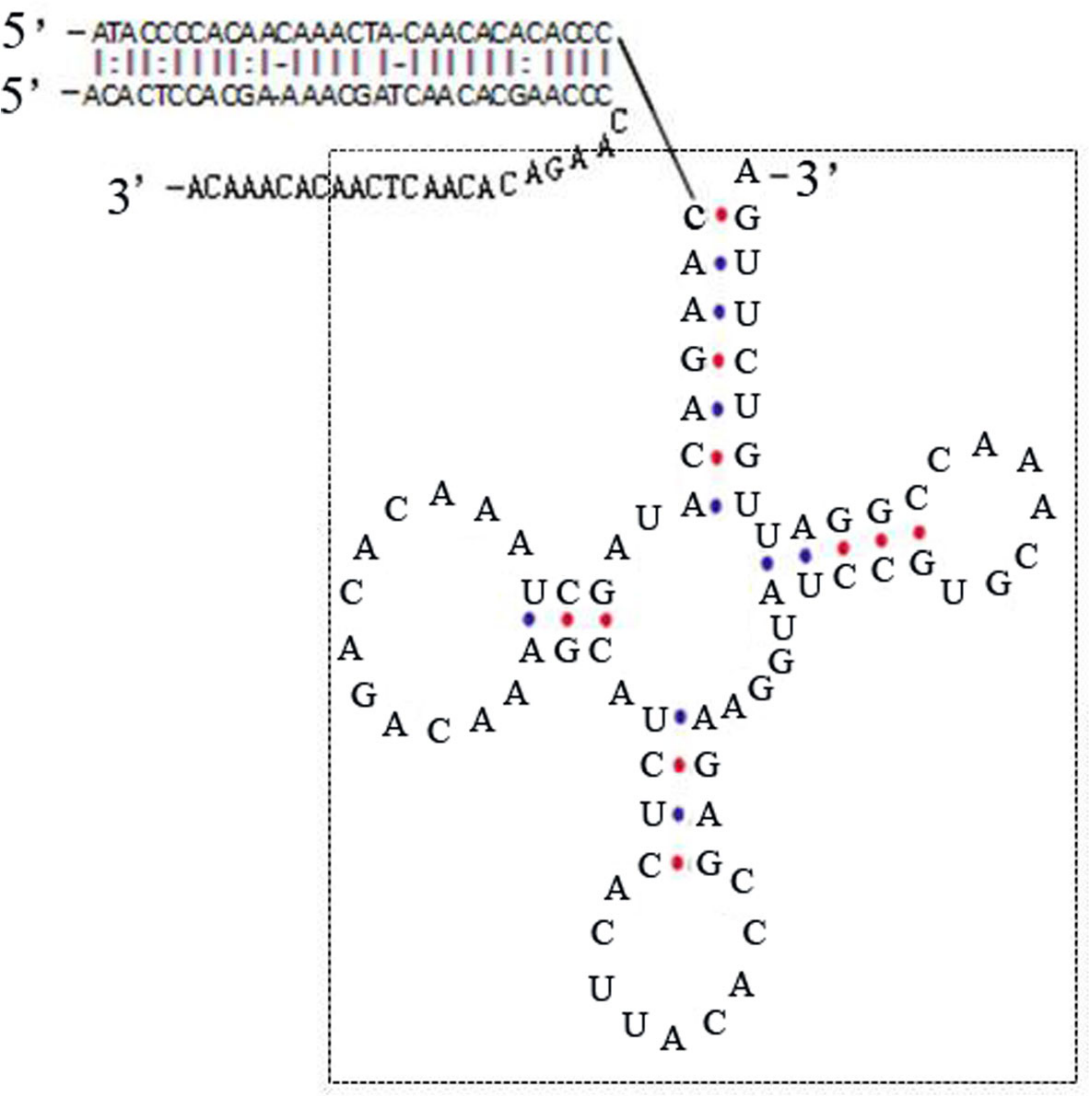
The cloverleaf secondary structures of the tRNA^Val^ gene in the mtDNA of *Iniistius trivittatus* and its flanking sequence are similar to the aligned sequences of tRNA-related retrotransposable elements from the red seaweed *Chondrus crispus*

In the known teleost mitochondrial genome, the 12S rRNA and 16S rRNA genes were separated by the tRNA^Val^ gene. However, two additional inserted noncoding regions (NCR1 and NCR2) were present in the *I. trivittatus* mtDNA; NCR1 was located between 12S rRNA and tRNA^Val^ and was 121 bp long, and NCR2 was situated between tRNA^Val^ and 16S rRNA and was 107 bp long. BLAST searches with algorithm parameters of word sizes from 32 to 16 did not retrieve any similar sequences. However, when using CENSOR to search for repetitive elements against the Repbase database, the results from the searches showed that NCR1 shares a 53-bp sequence with the internal portion of an LTR retrotransposon from the red seaweed *Chondrus crispus*. It is noteworthy that the common portions of the sequences composed the stem of the typical cloverleaf secondary structure of the tRNA^Val^ gene (Fig. 4); this finding leads to the hypothesis that the additional inserted noncoding sequences of mtDNA in *I. trivittatus* may be resident alien sequences resulting from the insertion of tRNA-related retrotransposable elements.

### Intraspecies and interspecies variation in additional insert sequences

To validate the two inserts in the *I. trivittatus* mtDNA, we sequenced a 988-bp long fragment of the mtDNA, including the 3′ end of the 12S rRNA gene, the complete tRNA^Val^ gene and the 5′ end of the 16S rRNA gene, from six individuals (GenBank Accession: MH198363–68). To analyze the sequence polymorphism, a complete Cyt b gene of 1141 bp was sequenced to be used as a reference (GenBank Accession: MH198369–74). The sequence alignment of the two inserts from the six individuals showed no difference in length and had 7 and 1 singleton variable sites (two variants) for NCR1 and NCR2, respectively. There is no parsimony-informative site for NCR1 or NCR2; for the Cyt b gene, 13 singleton variable sites and 4 parsimony-informative sites were found (Table 2).

**Table 2:** Polymorphism information for the additional noncoding insert regions (NCR1 and NCR2) and the Cyt b gene in the mtDNA of *Iniistius trivittatus*

N	Alignment position																								
	NCR1							NCR2	Cyt b																
																								1	1
	3	3	4	4	4	4	4	5				2	2	2	3	3	3	3	4	5	5	8	9	0	0
	5	9	2	3	3	3	4	4	6	6	8	1	2	9	0	3	4	9	4	2	9	8	4	9	9
	3	3	0	5	7	8	2	6	0	9	4	9	8	1	3	3	5	0	7	5	4	3	3	3	8
1	T	A	T	A	A	C	G	C	T	T	C	A	G	C	A	A	T	G	A	A	C	T	C	A	A
2	.	.	.	.	.	A	A	T	C	.	T	G	A	.	.	.	C	.	.	G	.	C	T	G	C
3	.	.	.	.	.	C	.	C	T	.	.	.	G	T	.	G	T	A	G	A	T	.	.	A	A
4	.	.	C	.	G	.	.	.	C	.	.	.	.	C	.	A	.	G	A	.	C	.	.	G	C
5	C	G	T	T	A	.	.	.	.	.	.	.	.	.	G	.	.	.	.	.	.	.	.	A	A
6	T	A	.	A	.	.	.	.	.	C	.	.	.	.	G	.	.	.	.	.	.	.	.	.	A

Note: N indicates the number of samples, and black dots indicate the same nucleotide

The region including the 3′ partial 12S rRNA gene, the complete tRNA^Val^ gene and the 5′ partial 16S rRNA gene in the mtDNA of *I. trivittatus* was used to detect the presence of NCR1 and NCR2 in species from the genus *Iniistius* (MH198358–71). The results from the comparison of three species (*I. trivittatus*, *I. dea* and *I. evides*) indicate that they share NCR1 and NCR2, but the length and similarity of these two regions are significantly variable (Table 3). For NCR1, the length varies from 115 bp in *I. dea* to 134 bp in *I. evides*, and the similarity is low and no more than 68%. For NCR2, the length varies from 96 bp in *I. dea* to 108 bp in *I. evides*, and the similarity is no more than 70% between the species. In contrast, the 12S rRNA, tRNA^Val^ and 16S rRNA genes were similar in length, and their sequences showed high similarity from 90.9% (16S rRNA in *I. trivittatus* vs. *I. dea*) to 100% (tRNA^Val^ in *I. trivittatus* vs. *I. dea*).

**Table 3:** Compositions and comparisons of five separate regions in mtDNA of *I. trivittatus* (I. tri), *I. evides* (I. evi) and *I. dea*

Region	Length (bp)			Identity (%)		
	*I.tri*	*I.evi*	*I.dea*	*I.tri - I.dea*	*I.tri - I.evi*	*I.dea - I.evi*
12S rRNA 3′ end	343	343	342	96.7	94.1	95
NCR1	121	134	115	61.3	67.7	65.1
tRNA-Val	74	74	74	100	98.6	98.6
NCR2	107	108	96	64.8	60.1	69.4
16S rRNA 5′ end	343	344	344	90.9	92.7	92.1

**Fig. 5:**
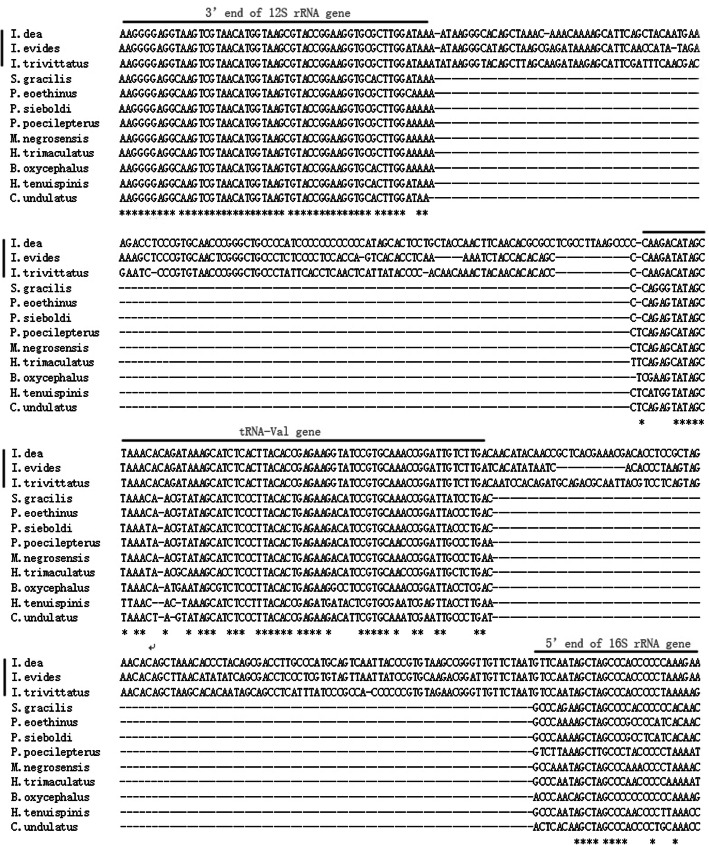
Sequence alignment of the mtDNA region from the 3′ end of the 12S rRNA gene to the 5′ end of the 16S rRNA gene including the complete tRNA^Val^ gene. The two inserts, one between the 3′ end of the 12S rRNA gene and the tRNA-Val gene and one between the tRNA^Val^ gene and the 5′ end of the 16S rRNA gene, are present in three species of the genus *Iniistius* (indicated by vertical lines) and are absent in the nine species of the other genera. Dashed lines indicate nucleotide indels, asterisks indicate conserved nucleotides, and lines above nucleotides indicate rRNA and tRNA gene boundaries

To date, approximately 8 species belonging to the following 6 genera in the labrid family have had their complete mitochondrial genome published in the NCBI database: *Bodianus*, *Cheilinus*, *Halichoeres*, *Macropharyngodon*, *Parajulis*, and *Pseudolabrus*. We did not find NCR1 or NCR2 in the mtDNA from the available data (Fig. 5). To further detect the presence or absence of the two inserts in other wrasses, we sequenced the portion of the mtDNA from the 3′ end of the 12S rRNA gene to the 5′ end of the 16S rRNA gene (including tRNA^Val^) in *S. gracilis*, and the inserts of NCR1 and NCR2 were lacking in this species (Fig. 5). The results based on the currently available datasets indicate that NCR1 and NCR2 specifically inserted into the mtDNA of species in the genus *Iniistius* and can serve as useful molecular markers for studies of mitochondrial evolution and for species identification.

### Phylogeny of the Labrida species

**Fig. 6:**
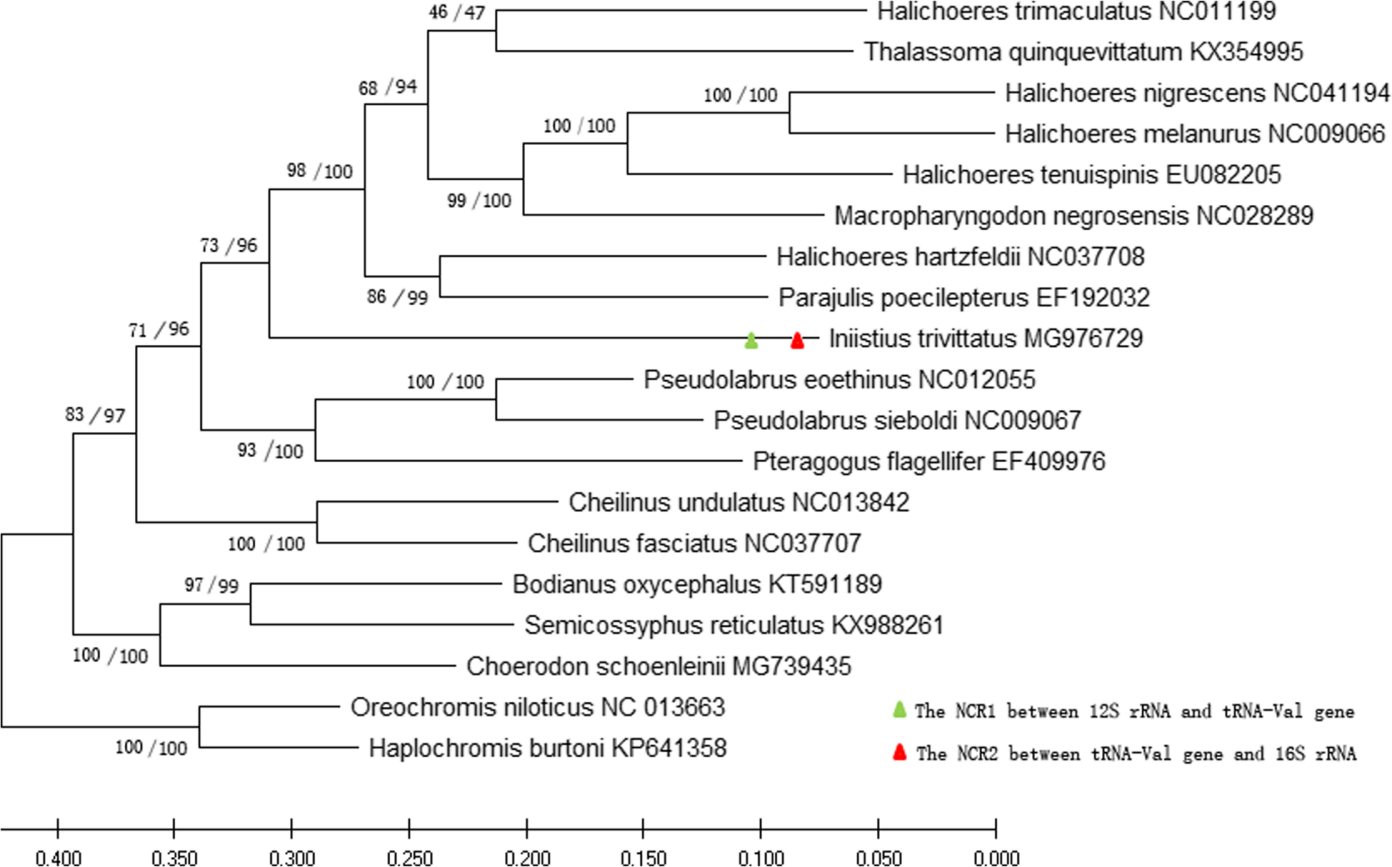
The cladogram of Labrida species based on the PCG dataset using ML and MP methods. Numbers on node indicated the bootsrap values of MP/ML tree in turn

The final combined PCG dataset had 10,546 characters after alignment, and there was no difference in topologies between the MP and ML tree of Labrida species (Fig. 6). The complete mitogenome dataset resulted in the phylogenetic trees from the MP and ML trees as same as the PCG topologies, respectively (data not shown). However, bootstrap values of the clades were higher in the PCG trees than the complete mitogenome trees. According to the phylogenetic results, *I. trivittatus* was recovered as monophyletic with strong supports bootstrap value 73% in MP tree, and 96% in ML tree. Two inserts, the NCR1 between 12S rRNA gene and tRNA-Val gene, and NCR2 between tRNA-Val gene and 16S rRNA gene were only observed in *I. trivittatus*.

## Discussion

In the present study, we describe a novel strategy to obtain accurate and complete sequences of the mitochondrial genome in wrasses. The first step was to obtain the sequences of the 16S rRNA, COI and Cyt b genes by PCR with universal primers [5, 22, 23], and the sequence gaps in these mitochondrial genes can then be obtained by the specific primers designed based on the known sequences listed above; the resulting fragments of the PCR products, obtained with only seven sets of PCR primers, are shorter than 3000 bp and can be sequenced by Sanger sequencing. Compared to other sequencing methods, such as PCR amplification with multiple PCR primer pairs (> 15) [21, 24] and next-generation sequencing [13, 19], our strategy can avoid nonspecific amplification or other problems resulting from sequence assembly.

We cloned the complete mitochondrial genome of *I. trivittatus* and found that the gene order of the mtDNA was as commonly described [21]. The gene length is similar to that of other wrasses; for example, the 12S rRNA gene lengths are 947 bp to 956 bp, and the 16S rRNA gene lengths are from 1684 bp to 1702 bp in the known mtDNA of eight species of wrasses (GenBank accession listed in Materials and Methods). The two rRNA genes in the mtDNA of *I. trivittatus* can form secondary structures similar to that of *P. eoethinus* (Additional Figs. 1 and 2). In addition, two novel inserted noncoding regions, NCR1 (in the middle of the 12S rRNA gene spanning to the tRNA^Val^ gene) and NCR2 (in the middle of the tRNA^Val^ gene spanning to the 16S rRNA gene), were found in *I. trivittatus* mtDNA. The two inserts have not been reported in previous studies of mtDNA, and furthermore, when we used the NCR1 and NCR2 sequences as query sequences to search against the NCBI database, no hits were retrieved. Notably, the 12S rRNA and 16S rRNA genes are highly conserved and invariant in fishes known to date [5, 6, 20, 21], and they showed little nucleotide site variation (1–9 bp) in invertebrate blacklegged ticks [25]. Therefore, NCR1 and NCR2 in the mtDNA of *I. trivittatus* did not exhibit the extensive variation of their flanking genes, 12S rRNA and 16S rRNA, as detected by sequence alignments and secondary structure analysis.

The most frequent foreign DNA insert in plant mitochondrial genomes, such as those in Alismatales, are transposable elements, which result in mtDNA size variation [19]. To identify the insertion origin in the mtDNA of *I. trivittatus*, we searched for repetitive elements against the Repbase Update database [26]. NCR1 is more similar to the internal portion of the LTR retrotransposable element from the red seaweed *Chondrus crispus*, and part of the matched sequences composed the stem of the typical cloverleaf secondary structures of the tRNA^Val^ gene (Fig. 4). This suggests that the inserts likely originate from tRNA-related retrotransposable elements. In the invertebrate cricket mitochondrial genome, a transposable element inserted into the 5′ flanking segments of the small rRNA gene and caused mtDNA length variation at the individual level [27]. In contrast, transposable elements can usually influence the integration of mtDNA in the nuclear genome, and thus, produce nuclear mitochondrial pseudogenes [28]. tRNA-containing insertions, which are extremely rare events, have been found in the mtDNA control region of species in the mussel family Mytilidae, and the insert origin can be explained by the occurrence of a tandem duplication, a nonhomologous recombination, or a deletion, because these mussels have two mtDNAs that are paternally inherited [29]. In our study, the mtDNA of *I. trivittatus* is maternally inherited, and the two inserts flanking the tRNA^Val^ gene were found to have no more than one repeat unit; the insert origin can be explained by tRNA-related retrotransposon insertion instead of nonhomologous recombination. The repeated block in the mtDNA control region of *I. trivittatus* (Fig. 3) resulted from a tandem duplication.

To understand the population structure and genetic variability at the intraspecies level of the two inserts, NCR1 and NCR2, we designed primers corresponding to the conserved block of the 12S rRNA and 16S rRNA genes to PCR amplify the spanned sequences between the 3′ end of the 12S rRNA gene and the 5′ end of the 16S rRNA gene, including NCR1, the complete tRNA^Val^ gene, and NCR2. We detected six individuals of *I. trivittatus* by PCR with the primers, and the insert sequences were highly conserved at the intraspecies level (Table 2). Compared with the Cyt b gene, the number of singleton variable sites of the inserts is lower (7 for NCR1, 1 for NCR2, and 13 for Cyt b), and parsimony-informative sites are lacking (4 for Cyt b), which revealed evidence of low genetic variability in NCR1 and NCR2. On the other hand, parsimony-informative sites were observed in the Cyt b gene, suggesting that the Cyt b gene has undergone changes and that a few silent mutations allow enough population differentiation. A hypothesis regarding transposon insertion mutations is that transposon insertion into the host genome can cause a mutation, including insertion, deletion, and nucleotide mutations, of the host genome and then lead to genetic homoplasmy in the population by natural selection [30]. Overall, this hypothesis can be employed to explain the occurrence of the genetic invariability of NCR1 and NCR2 and the variability of the Cyt b gene in the mtDNA.

Analysis of sequence alignments of NCR1 and NCR2 in the razorfishes *I. trivittatus*, *I. evides* and *I. dea* at the interspecies level revealed that their lengths vary and that they have low similarity (< 70%) among the species, while the tRNA^Val^ gene situated between NCR1 and NCR2 showed high similarity among the species (98.6–100%) (Table 3). The sequences of NCR1 and NCR2 are variable at the interspecies level and are conserved, providing evidence for the hypothesis of transposon insertion mutations [30]. The sequence variation of NCR1 and NCR2 in razorfishes suggests that these regions of the mtDNA have potential as a useful genetic marker for examining the genetic structures, classification and evolutionary history of many species of razorfish; razorfishes, as coral reel fishes, are diversified in color and size, and some species were misidentified due to their changes in color [2]. Thus, the markers NCR1 and NCR2 provide an additional assessment of the genetic diversity of razorfishes.

The NCR1 and NCR2 present in razorfishes are absent in other wrasses based on NCBI data analysis (8 species in 6 genera: *Bodianus*, *Cheilinus*, *Halichoeres*, *Macropharyngodon*, *Parajulis* and *Pseudolabrus*) and PCR verification (1 species in 1 genus: *Suezichthys*) (Fig. 5). In a study on the evolutionary origins of wrasses, Cowman et al. [31] reconstructed the origin and diversification of the Labridae using two mitochondrial genes and two nuclear protein-coding genes and showed that the phylogenetic relationships among these genera can be reconstructed as follows: ((((*Bodianus*), *Cheilinus*, *Halichoeres*/ *Macropharyngodon*), *Iniistius*), *Pseudolabrus*) [31]. Interestingly, only razorfish species of the genus *Iniistius* have NCR1 and NCR2, although the divergence of *Iniistius* is early relative to *Pseudolabrus*. Data from our study indicate rare inserts of transposable elements in the mtDNA of ancient razorfishes, and multiple origins caused the emergence of most of the major wrasse genera due to their novel feeding modes. To obtain the evolutionary history with the insert events about NCR1 and NCR2, we checked the phylogenetic trees based on the complete mitogenome dataset, and on the protein-coding gene dataset of Labrida species, respectively. According to the evolutionary topologies, *I. trivittatus* was monophyletic with strong support’s bootstrap value, and the insert events were only found in this species, which was consistent with published phylogenies that the extant lineages in Labrida species show most diversification (31). Our results confirm that the specialized feeding modes (coral feeding, foraminifera feeding and fish cleaning) in wrasses drove their evolution and led to their biodiversity [7]. In the case of the species in *Iniistius*, *I. trivittatus*, first identified by Randall & Cornish [32], it is only known from deep outer reef habitats, where they occur in small, loose groups along sand ridges. *I. dea* always dive into the sand to search for small animals under the sand, while *I. evides* lives in sandy, open areas near shallow reefs at approximately 5 m [9]. It is reasonable to think that environmental pressure may have led to the sequence variation in NCR1 and NCR2 in the mtDNA of razorfishes observed at the interspecies level. It would be interesting to use these inserts as evolutionary markers to widely investigate their effects on the evolution of the mitochondrial genome in wrasses.

## Conclusions

Wrasses have a multiple origin and independent evolutionary history of the family was influenced by feeding modes. Comparable studies using mitochondrial sequences with divergence dates for multiple taxa provided an evidence that two rare inserts in the intergenic regions 12S rRNA - tRNA^Val^ - 16S rRNA are highly conserved at the intraspecies level, and significant variation at the interspecies level. These insert events will be important in understanding species divergence and mitochondrial evolution in wrasses.

## Methods

### Sample collection and DNA extraction

Fish samples of 7 individuals from *I. trivittatus*, 3 individuals from *I. dea*, 1 individual from *I. evides*, and 1 individual from *Suezichthys gracilis* were dead before sample collection and were purchased from the Huangsha aquatic product Market in Guangzhou Province near the South China Sea in China. Tissue samples from these individuals were preserved in 95% ethanol and stored at − 80 °C before use. The relevant specimens were kept in the Laboratory of Ichthyology, Shanghai Ocean University, China. Total DNA was isolated from the tissue samples using proteinase K digestion in lysis buffer at 55 °C for 2–3 h, following the protocols of the manufacturer of the UNIQ-10 DNA Extraction Kit (Sangon, Shanghai, China).

### PCR amplification strategy and sequencing

The complete mitochondrial genome of *I. trivittatus* was amplified by a novel strategy that included the following steps: 1) three sets of fish-universal primers of the 16S rRNA [5], COI [22], and Cyt b [23] genes were used to amplify the sequences in the corresponding regions in *I. trivittatus*; 2) to obtain the gap sequences of 16S rRNA to COI, tRNA-Leu to Cyt b, and Cyt b to 16S rRNA, three sets of primers were designed based on the above sequenced regions and were used to amplify the gap regions; and 3) a set of primers designed based on the sequences of the COI, tRNA-Leu and Cyt b genes were used to amplify the gap between the COI and tRNA-Leu genes (Additional Table 1). A total of seven sets of primers were used to amplify the entire mitochondrial genome of *I. trivittatus*, and the fragments of PCR products were usually short enough to be sequenced by Sanger sequencing. The PCRs were carried out in 25 μl reaction mixtures containing 12.5 μl 2X Taq PCR Master Mix, 0.5 μl primers (10 μM each), 11 μl distilled water, and 0.5 μl of DNA template (~ 100 ng). The PCR amplification conditions were as follows: 94 °C for 2 min, 35 cycles of denaturing at 94 °C for 45 s, annealing at 58–60 °C for 55 s, and extension at 72 °C for 1 min (before steps 1 and 3) or 3 min (before step 2); and a final extension for 10 min at 72 °C. The PCR products were evaluated by 1.5% agarose gel electrophoresis and sequenced by Sangon Biotech Co., Ltd. (Shanghai, China).

### Sequence assembly and analysis

Raw sequences were assembled into a complete mitochondrial genome using BioEdit [33] and annotated using MitoFish [34]. The thirteen protein-coding genes were verified by BLAST and ORF Finder (Open Reading Frame Finder: https:// www. ncbi.nlm.nih.gov/orffinder/); the start and stop positions of the 2 rRNAs were identified by sequence comparison with other mtDNA in the NCBI database and then verified by secondary structure prediction using Mfold with the default settings [35]. All tRNA secondary structures were predicted using tRNAscan-SE [36]. The noncoding regions were identified by sequence homology analysis. The base composition and skew of the complete mtDNA were calculated using MEGAX version 10 [37]. The transposable elements were identified with CENSOR software by searching against the Repbase database [26]. The analysis of sequence polymorphisms was performed using DnaSP 6.0 [38]. Finally, all the sequences from different individuals and species were aligned using ClustalX [39].

### Labrid mitochondrial gene size comparisons

We downloaded the mitochondrial genome sequences of *Pseudolabrus sieboldi* (GenBank Accession AP006019) [6], *Pseudolabrus eoethinus* (GenBank Accession EU560728), *Halichoeres trimaculatus* (GenBank Accession EU087704), *Halichoeres tenuispinis* (GenBank Accession EU082205), *Macropharyngodon negrosensis* (GenBank Accession KP013102), *Parajulis poecilepterus* (GenBank Accession EF192032) [20], *Bodianus oxycephalus* (GenBank Accession KT591189), and *Cheilinus undulatus* (GenBank Accession KM461717) [21], which had been deposited as complete genomes in the NCBI database, to align mitochondrial gene sizes, including 12S rRNA, tRNA^Val^, and 16S rRNA. The sequences of this region spanning 12S to 16S rRNA in the mtDNA of *I. dea*, *I. evides*, and *S. gracilis* were obtained by PCR with a set of primers (named 12-16S) designed based on the sequence of *I. trivittatus* (Additional Table 2), and the PCR conditions were as described above except the annealing temperature of 57 °C. A locus site of the complete Cyt b gene in the mtDNA was used as the reference in the polymorphism analysis of the two inserts in *I. trivittatus* with a set of the designed primers (Additional Table 2). The conserved domain and accompanying motif annotations of the mitochondrial control region were visualized as described [40].

### Phylogenetic analyses of Labridae

In Labridae, 17 fish mitogenomes are available in GenBank and publications (before April 28, 2020) [6, 20, 21, 41], and two fish mitogenomes of Cichlid species, *Oreochromis niloticus* and *Haplochromis burtoni* were acted as outgroups (all GenBank Accession numbers were shown in Fig. 6). Datasets containing 13 protein- coding genes (PCG), 22 tRNA genes, and two rRNA genes plus the control region were used to investigate the phylogenetic relationships within Labridae. The evolutionary analysis of Labrida species was separately performed with a combined PCG dataset including ND1, ND2, ND3, ND4L, ND4, ND5, ND6, COI, COII, COIII, ATP8, ATP6 and Cyt b, and their first, second and third codons. The nucleotide sequences were aligned using ClustalW, and phylogenetic analysis was performed using MEGAX version 10 (37) for the Maximum parsimony (MP) and maximum likelihood (ML) methods. For ML analyses, the model GTR was chosen for the likelihood analyses. The reliability of the clades in the phylogenetic trees was assessed by bootstrap probabilities computed using 1000 replicates. The 1000 replicates bootstrap support was also performed in the MP analysis.

The events of inserts onto the phylogenetic trees were checked using a combined strategy, 1) sequences of the NCR1 and NCR2 were aligned with the mitogenome of individual species to find their similarities; 2) the conserved regions at the 3′ end of 12S rRNA gene and the 5′ end of tRNA-Val gene in the individual mitogenome were used to check present/absent of the NCR1; the conserved regions at the 3′ end of tRNA-Val gene and the 5′ end of 16S rRNA gene in the individual mitogenome were used to check present/absent of the NCR2.

## Supplementary information

**Additional file 1: Figure S1.** The secondary structure of the 5′ end, 400 bp length, of the mitochondrial 16S rRNA gene for *Pseudolabrus eoethinus*.

**Additional file 2: Figure S2.** The secondary structure of the 5′ end, 400 bp length, of the mitochondrial 16S rRNA gene for *Iniistius trivittatus*.

**Additional file 3: Table S1.** Primers used to amplify the complete mitochondrial genome sequences of *Iniistius trivittatus*. **Table S2.** Primers used to amplify the region, including 12S rRNA (partial), two additional inserts, tRNA-Val and 16S RNA genes by the 12–16 primer set; the complete cytochrome b gene was amplified by the Cyt b primer set.

## Data Availability

DNA sequence data generated and analysed in this manuscript are deposited in a public database, NCBI. Accession numbers can be found in contents.

## Abbreviations

16S rRNA: 16S ribosomal RNA
12S rRNA: 28S ribosomal RNA
mtDNA: Mitochondrial DNA
TAS: Termination associated sequence
CSB: Central conserved sequence blocks
NCR: Additional inserted noncoding regions
