# Supplementary figures and images for: Complete mitochondrial genome of Iniistius trivittatus and unique variation in two observed inserts between rRNA and tRNA genes in wrasses
Source: BMC Evol Biol. 2020 Sep 21;20:125. doi: 10.1186/s12862-020-01683-8 (PMC7507615; doi:10.1186/s12862-020-01683-8)

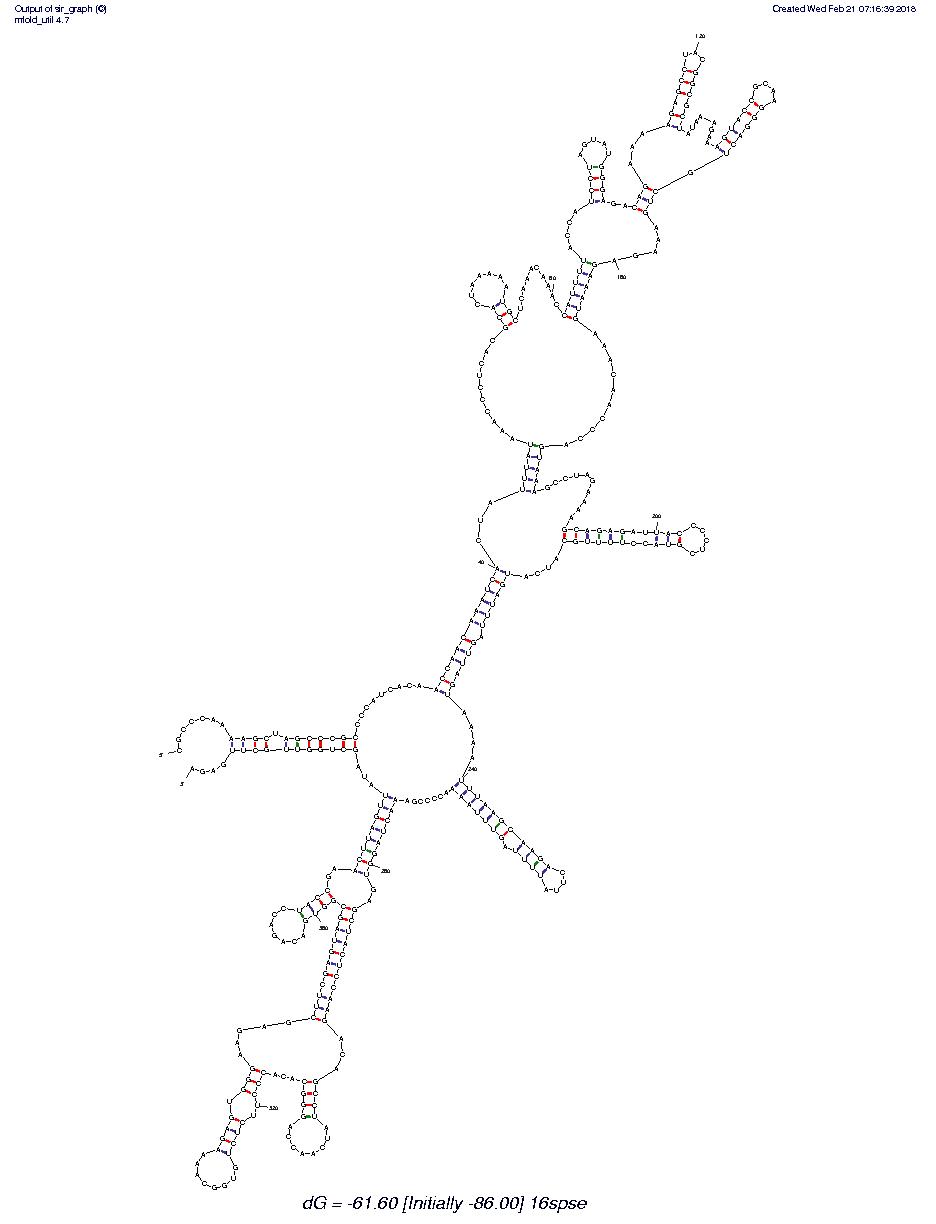

Supplement: Supplementary file 1 — Additional file 1: Figure S1. The secondary structure of the 5′ end, 400 bp length, of the mitochondrial 16S rRNA gene for Pseudolabrus eoethinus. [file 12862_2020_1683_MOESM1_ESM.jpg]

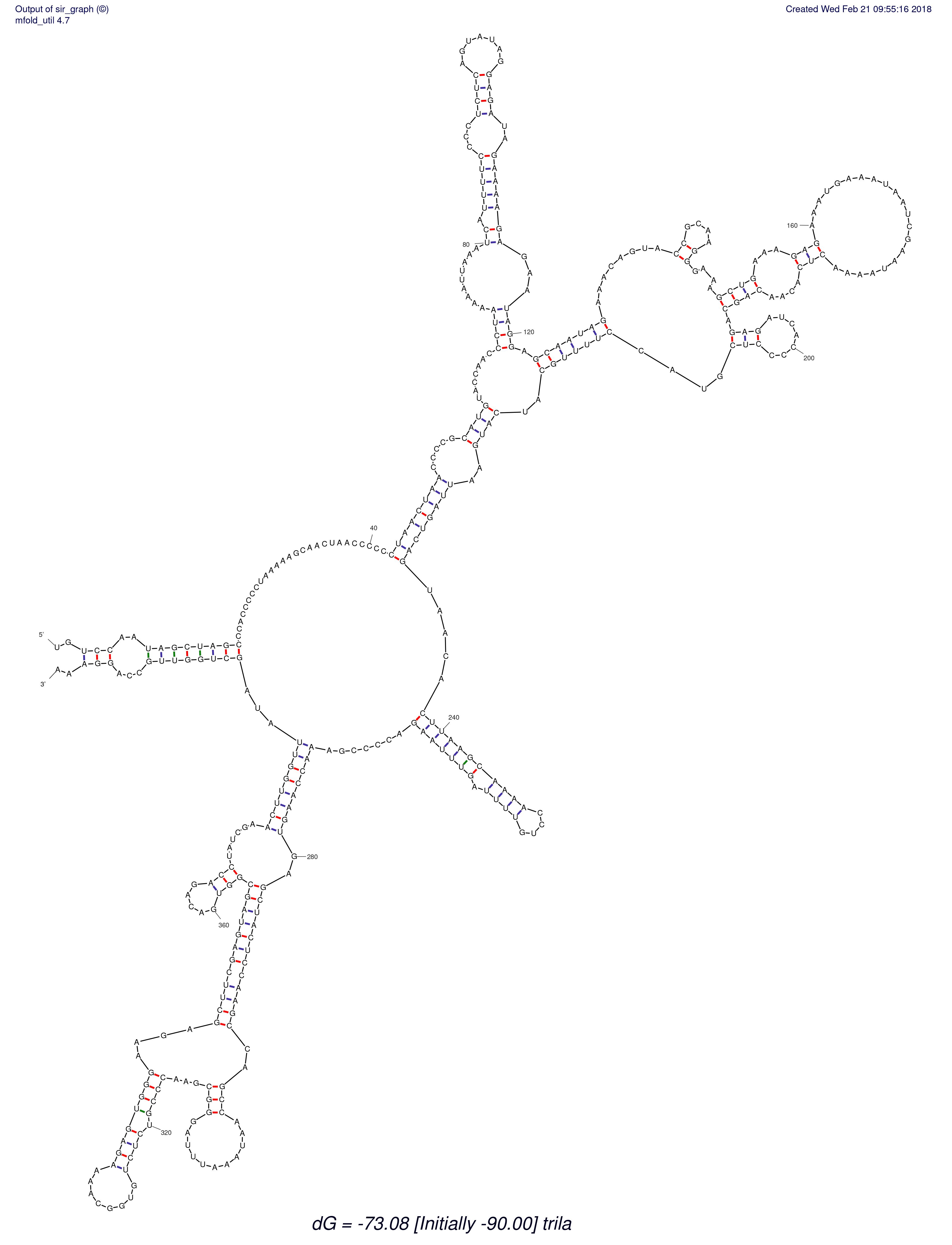

Supplement: Supplementary file 2 — Additional file 2: Figure S2. The secondary structure of the 5′ end, 400 bp length, of the mitochondrial 16S rRNA gene for Iniistius trivittatus. [file 12862_2020_1683_MOESM2_ESM.jpg]
