# Supplementary material for: Complete mitochondrial genome of Iniistius trivittatus and unique variation in two observed inserts between rRNA and tRNA genes in wrasses
Source: BMC Evol Biol. 2020 Sep 21;20:125. doi: 10.1186/s12862-020-01683-8 (PMC7507615; doi:10.1186/s12862-020-01683-8)
Supplement: Supplementary file 3 — Additional file 3: Table S1. Primers used to amplify the complete mitochondrial genome sequences of Iniistius trivittatus. Table S2. Primers used to amplify the region, including 12S rRNA (partial), two additional inserts, tRNA-Val and 16S RNA genes by the 12–16 primer set; the complete cytochrome b gene was amplified by the Cyt b primer set. [file 12862_2020_1683_MOESM3_ESM.docx]

Additional table 1 Primers are used to amplify the complete mitochondrial genome sequences of *Iniistius trivittatus*

| Primer name | Sequence(5→3) |
| --- | --- |
| 16S Forward primer | AGAGAAAGTACCGCAAGGGAAAGC |
| 16S Reverse primer | TCCTGATCCAACATCGAGGTCGTA |
| CO1 Forward primer | GGCTACAACCCACCGCTTAAACC |
| CO1 Reverse primer | AGTCTGAGTATCGTCGAGGCATTCC |
| Cytb Forward primer | ACCACCGTTGTTATTCAACTACAAGAAC |
| Cytb Reverse primer | CCGACTTCCGGATTACAAGACCG |
| 16S-COI Forward primer | AAGCAGATATGTTAATCACCTCCTACAGAG |
| 16S-COI Reverse primer | GCCGAAGAATCAGAATAAGTGTTGGTAG |
| tRNA-Cytb Forward primer | ACAGCTCATCCGTTGGTCTTAGG |
| tRNA-Cytb Reverse primer | GAGGTGTAGTGTATGGCGAGGAA |
| Cytb-16S Forward primer | CCTCAGTCCTGTACTTCTTCCTCTTC |
| Cytb-16S Reverse primer | CGATAGGTCTGTCACCGCTACTC |
| COI- tRNA Forward primer | TTTCAAGCCAACCACATAAC |
| COI- tRNA Reverse primer | CGACCCCTTCCCAGCCAATA |

Additional table 2 Primers were used to amplify the region including 12S rRNA partial, two additional inserts, tRNA-Val and 16S RNA genes by 12-16 set; the complete cytochrome b gene was amplified by Cytb set.

| Primer name | Sequence(5→3) |
| --- | --- |
| 12-16S Forward primer | GATCCACCTAGAGGAGCCTGTTC |
| 12-16S Reverse primer | CTTCTATCCATCTTCCAAGCAACCA |
| Cytb Forward primer | CGGATTCCAACCAGGACTTACGA |
| Cytb Reverse primer | GGGAGTTAGTGGTGGGAGTTAGAG |
